# Human Metapneumovirus Circulation and Seasonality on a Global Scale, 2016–2025: Changes in Patterns and Epidemic Timing in the Pre‐ Versus Post‐COVID‐19 Era

**DOI:** 10.1111/irv.70200

**Published:** 2025-12-17

**Authors:** Enrica Castellana, Alessandra Picelli, Emma Papini, Guglielmo Bonaccorsi, Jojanneke van Summeren, Marco Del Riccio, Saverio Caini

**Affiliations:** ^1^ Department of Health Sciences University of Florence Florence Italy; ^2^ Netherlands Institute for Health Services Research (Nivel) Utrecht the Netherlands; ^3^ University of Florence Florence Italy

**Keywords:** epidemics, human metapneumovirus, public health strategies, seasonality, surveillance, timing

## Abstract

**Background:**

Human metapneumovirus (hMPV) circulates globally, yet its seasonal dynamics remain incompletely defined. Here, we describe the global timing, amplitude and duration of hMPV outbreaks, comparing the pre‐ versus post‐COVID‐19 periods.

**Methods:**

Surveillance data for hMPV were retrieved from WHO FluNet from Week 1/2016 until Week 26/2025. We examined the epidemic peak timing, amplitude and duration across seasons and latitudes and compared patterns in 2016–2019 with those in 2021–2025.

**Results:**

Over the study period, approximately 145,000 hMPV detections were reported to the WHO FluNet database from a total of 54 countries worldwide. Among the 15 countries with sufficient data to analyse seasonality, the epidemic timing aligned with geographic latitude, with the peak occurring in June–September in the Southern Hemisphere, February–April in the Northern Hemisphere, while the timing was variable across the intertropical belt. The amplitude of the peak varied across countries, with some countries characterized by single, well‐defined peaks and others with more widespread epidemics throughout the season. The median epidemic duration was 19.5 weeks (range 15–36). After the appearance of SARS‐CoV‐2, marked shifts in timing and amplitude were observed, with delays or dislocations in several countries compared with pre‐pandemic seasons.

**Conclusions:**

In this analysis of global surveillance data for hMPV (extended until June 2025), we highlighted latitudinal gradients in hMPV circulation, with disruptions associated with COVID‐19. Our findings emphasize the importance of sustained, type‐specific global surveillance to inform public health strategies and to characterize the post‐COVID‐19 global seasonality patterns of hMPV.

## 1 Introduction

Human metapneumovirus (hMPV) is a respiratory pathogen belonging to the Pneumoviridae family, genus Metapneumovirus, first identified in the Netherlands in 2001 in children with acute respiratory infections, although its circulation has been documented since the 1950s [1]. It is a single‐stranded, non‐segmented, negative‐polarity RNA virus, approximately 13 kb in length, which encodes nine structural proteins and has two main genotypes (A and B). Genotypes A and B are further divided into two subgroups each (A1, A2 and B1, B2) [2, 3]. From a clinical point of view, hMPV is responsible for a wide range of manifestations, from common upper respiratory tract infections to severe forms of bronchiolitis and pneumonia, particularly in young children, the elderly and immunocompromised individuals [4]. Coinfection with other respiratory pathogens, such as respiratory syncytial virus (RSV) and influenza, is common and contributes to a worse prognosis [5, 6]. Epidemiological studies estimate that hMPV is responsible for 5%–10% of paediatric hospitalizations for acute respiratory infections, with clinical manifestations often indistinguishable from those caused by RSV [7, 8]. In adults, hMPV is increasingly recognized as a relevant cause of acute respiratory disease, being associated with substantial morbidity and excess hospitalizations; in older adults, hMPV is estimated to cause approximately 9–10 hospitalizations per 10,000 persons annually, underscoring its substantial clinical burden [9]. Currently, there are no specific antivirals or approved vaccines against hMPV; treatment is mainly supportive [3, 7, 8]. The development of combination vaccines including RSV, hMPV and human parainfluenza virus 3 (HPIV3) is of growing interest in the scientific community, as this approach could broaden immune protection and contribute significantly to reducing the global burden of respiratory infections [10].

Despite its clinical relevance, hMPV continues to be underdiagnosed and underreported, as its clinical manifestations frequently overlap with those caused by RSV, influenza viruses or other respiratory pathogens [3]. In past years, reverse transcriptase polymerase chain reaction (RT‐PCR) has become widespread and is currently the gold standard for the detection of hMPV and other respiratory viruses in clinical specimens, both for clinical purposes and for epidemiological and virological surveillance, thanks to its high sensitivity and specificity [11, 12]. In contrast, viral isolation has significant practical limitations due to the slow replication of the virus, which hinders its application in routine diagnostics. The key notable advantage of real‐time PCR lies in its ability to support multiplexing, as the use of probes labelled with different fluorophores allows for simultaneous discrimination of multiple amplified targets, which contributes to both time and cost efficiency [4, 12].

Given the absence of a mandatory reporting requirement, global virological surveillance systems like WHO FluNet are essential for outlining hMPV epidemiology. Within this framework, a deep understanding of the virus's seasonality is paramount, as it directly informs crucial public health actions, from optimizing surveillance to preparing for annual outbreaks. This knowledge has gained further importance with vaccines on the horizon, where defining clear circulation patterns is critical for timing future immunization campaigns to maximize their effectiveness. The recent COVID‐19 pandemic caused an unprecedented disruption to established viral dynamics, creating significant uncertainty and an urgent need for updated evidence. Our study addresses this gap by exploiting global surveillance data to characterize hMPV seasonality before and after the pandemic, with the ultimate aim of supporting the development of more resilient and effective prevention strategies.

## 2 Methods

### 2.1 Data Sources Definitions

Weekly counts of laboratory‐confirmed hMPV detections were retrieved from the WHO FluNet platform (https://www.who.int/tools/flunet) on 15 July 2025, with data up to epidemiological Week 26 of 2025. Countries were classified into three broad latitude‐based regions depending on the coordinates of their geographic centroid: Northern Hemisphere (north of the Tropic of Cancer), Southern Hemisphere (south of the Tropic of Capricorn) and the intertropical belt. Because respiratory virus seasonality varies by latitude, we defined ‘season’ differently across geographic zones, aligning calendar boundaries with the expected timing of epidemic activity in each hemisphere. A ‘season’ was made to overlap with the calendar year (Weeks 1–52/53) for intertropical belt and Southern Hemisphere countries, whereas for Northern Hemisphere countries the ‘season’ was defined as extending from Week 27 of 1 year to Week 26 of the following year [13]. Following this definition, surveillance data from Week 1/2016 to Week 26/2025 allowed the reconstruction of up to nine complete seasons: 2016–2017 through 2024–2025 for the Northern Hemisphere and 2016–2024 for the intertropical belt and the Southern Hemisphere. To avoid including incomplete seasons, Weeks 1–26/2016 for the Northern Hemisphere and Weeks 1–26/2025 for the intertropical belt and Southern Hemisphere were excluded. In what follows, the unit of analysis was the ‘country‐season’, defined as data for a given season in a given country.

At present, the WHO FluNet platform does not allow the computation of hMPV positivity rates, as the denominator (i.e., the number of specimens tested for hMPV) is not reported. Consequently, the present study concentrated on the seasonal dynamics of hMPV activity, specifically focusing on the timing of epidemic peaks and the overall duration of epidemic periods.

In FluNet, country‐level data are classified into three categories:

- Sentinel surveillance: routinely collected information from structured sentinel systems, most often a network in the primary care setting.
- Nonsentinel surveillance: data derived from outbreak investigations, universal or point‐of‐care testing or other sources outside sentinel surveillance.
- Not defined: entries without a clear classification, which may represent a mixture of sentinel and nonsentinel data.

For certain countries and seasons, multiple sources of surveillance data were available. Although there is limited biological or epidemiological rationale to expect major differences in epidemic timing or duration by case severity, minor variations across surveillance systems are possible. For example, differences in transmission dynamics, age distribution and healthcare‐seeking behaviour may lead to modest discrepancies between sentinel and SARI surveillance, as occasionally observed for other respiratory viruses such as RSV. We adopted the strategy of selecting, for each country‐season, the surveillance system type that provided the largest number of hMPV detections, as done in previous studies [13]. This choice was made to maximize statistical power and to obtain more robust estimates of epidemic timing. Before this step, however, country‐seasons with fewer than 30 reporting weeks were excluded, irrespective of the number of detections or surveillance system type, to minimize the risk of incomplete epidemic coverage and consequent bias in estimating the peak timing and epidemic length.

### 2.2 Statistical Analysis

We compiled the number of hMPV detections reported to FluNet for each country‐season and categorized country‐seasons into three groups: 1–24, 25–49 and ≥ 50 detections per season. Country‐seasons with no detections were excluded, as the possibility of absent testing could not be ruled out. We then summarized the distribution of these categories according to latitudinal zone, WHO region and season.

The seasonal dynamics of hMPV circulation were assessed through country‐specific time series analyses performed with the EPIPOI software (https://www.epipoi.info/) [14]. The 2020 season (2020–2021 for Northern Hemisphere countries) was excluded from this specific analysis on seasonality due to its atypical nature linked to the COVID‐19 pandemic. Our primary goal was to characterize global hMPV epidemic patterns and compare pre‐pandemic and postpandemic periods. To strengthen reliability, only countries reporting ≥ 50 detections in at least three seasons, either before or after the pandemic (or in both periods), were included in the analyses (country‐seasons with fewer than 50 detections were excluded upfront). EPIPOI first detrends the time series by fitting a quadratic polynomial and then reconstructs the periodic annual function (PAF) through Fourier decomposition, incorporating annual, semi‐annual and quarterly harmonics. The timing of the epidemic peak is defined as the month corresponding to the maximum of the PAF, while the peak amplitude is expressed as the ratio between wave height and peak value, presented as a percentage. Amplitude quantifies the degree of clustering of cases around the typical seasonal peak, and by construction, it may occasionally exceed 100% [14]. Peak timing and amplitude were initially estimated for each country over the entire study period (2016–2025) and then recalculated separately for the pre‐pandemic and postpandemic periods for those countries with sufficient data (≥ 3 qualifying seasons in both periods). Lastly, epidemic duration for each country‐season was derived using the 75% average annual percentage (AAP) method [15], which identifies the shortest sequence of consecutive weeks accounting for at least 75% of annual detections.

### 2.3 Software

All statistical procedures were performed with Stata Version 17 (StataCorp, College Station, TX, USA) and with the open‐access software EPIPOI (https://www.epipoi.info/).

## 3 Results

### 3.1 Data Availability and Descriptive Summary

Over the study period, approximately 145,000 hMPV detections were reported to FluNet by 54 countries and territories, accruing data for a total of 264 country‐season combinations. The contribution of individual countries exhibited considerable heterogeneity: the median number of detections per country‐season was 49, with approximately 40.9% (*n* = 108) of seasons reporting fewer than 25 detections, while 50.0% (*n* = 132) reported 50 or more detections. A summary of hMPV detections by latitude, WHO region and season is available in Tables S1–S3. The median number of hMPV detections per country‐season varied across latitude‐defined areas, as it was 24 in the intertropical belt, 137 in Northern hemisphere countries and 801 in Southern hemisphere countries (Table S1). Regional analysis, based on WHO classifications, further underscored this heterogeneity (Table S2). The Americas region accounted for the largest share of detections (100,643; median 38 per country‐season), followed by the Western Pacific (34,148; median 89) and the Eastern Mediterranean (8265; median 94). Conversely, data availability remained severely limited in Africa and Southeast Asia, while there was no reporting at all from the WHO European Region in FluNet. The annual total number of detections demonstrated significant fluctuations as well. A nadir was observed in 2020, with only 919 reported detections (Table S3). Subsequently, a consistent increase was noted from 2022 to 2024, culminating in a substantial rise in detections reaching 33,814 in 2024. Concurrently, the median number of detections per country‐season mirrored this trend, rising from 9 in 2020 to 90 in 2024.

### 3.2 Peak Timing and Duration of hMPV Epidemics

Based on the predefined cut‐offs detailed in the methodology, the temporal characteristics of hMPV epidemics (month of peak incidence, amplitude and duration) were ascertainable for 15 of the 54 countries that reported at least one detection during the study period. The remaining 39 countries were excluded from the seasonality analyses because they did not meet the predefined completeness threshold (i.e., fewer than three seasons with ≥ 50 detections). The country‐specific time series for these countries is provided in Figures S1–S15.

**FIGURE 1:**
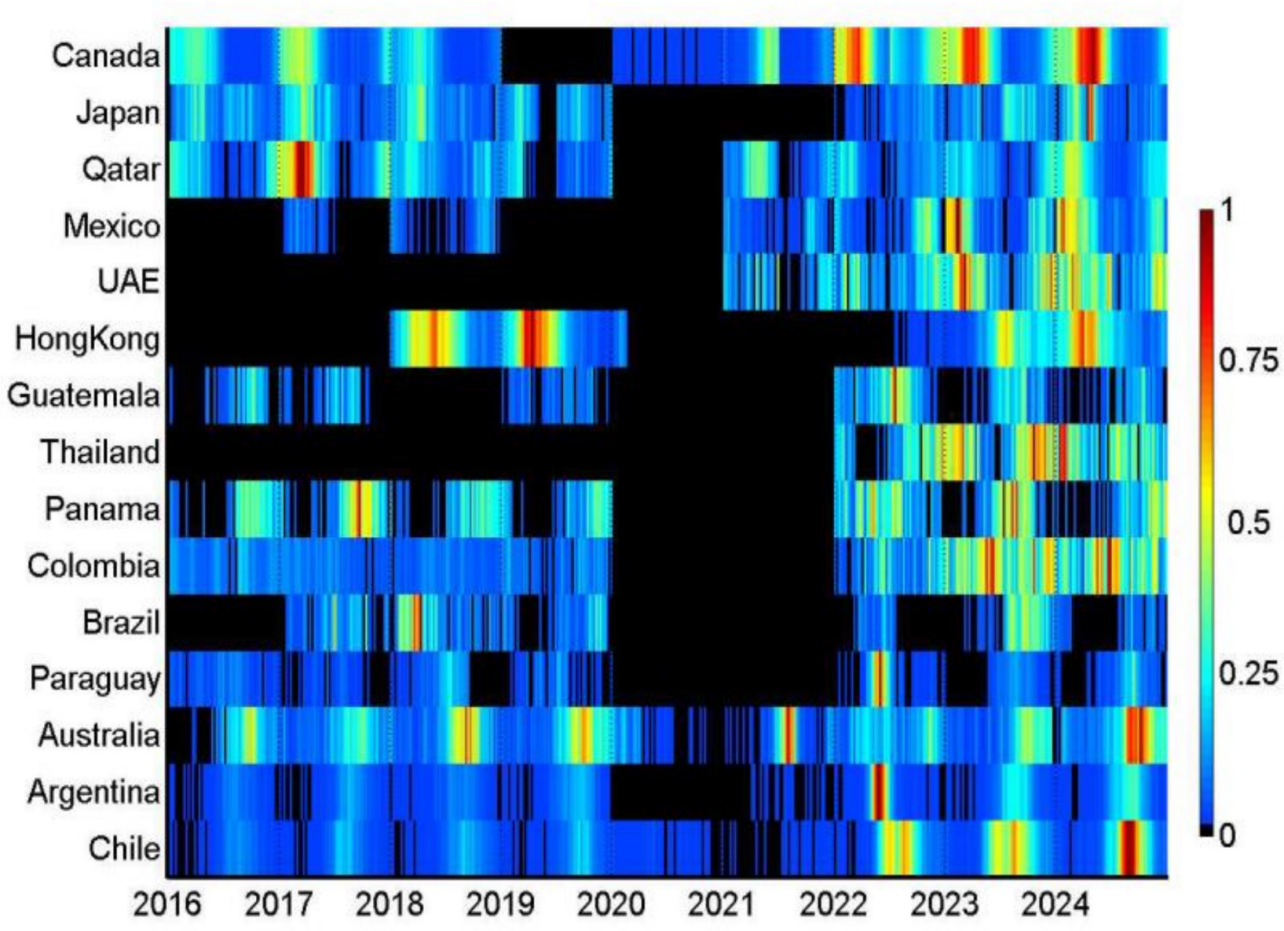
Heatmap of hMPV detections by country and month. Colours represent the relative monthly intensity of detections within each country and period, scaled from low (blue) to high (red).

**FIGURE 2:**
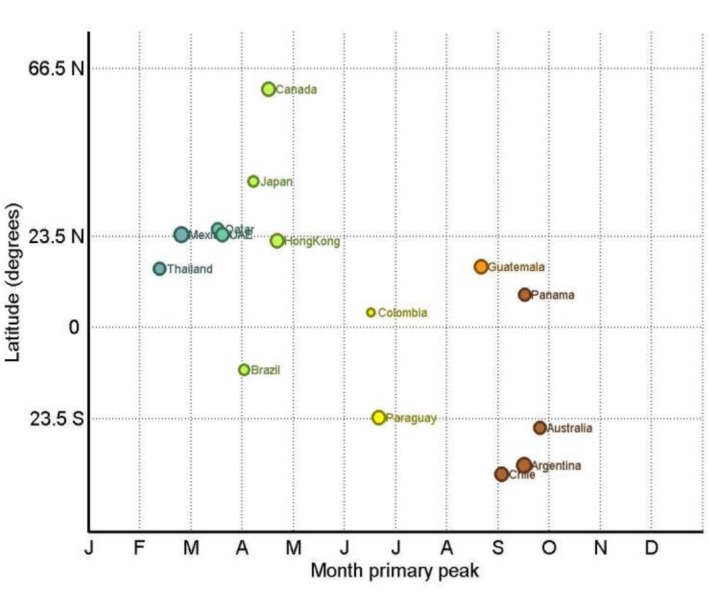
Typical timing of the primary peak of hMPV epidemics by country latitude. The diameter of the circle is proportional to the peak amplitude (see text for details). WHO FluNet, 2016–2025.

Remarkable differences in peak timing were observed in relation to latitude, with certain countries deviating from the predominant hemispheric trends (Table 1 and Figures 1 and 2). In Southern Hemisphere countries, peaks predominantly occurred during autumn, with Chile, Argentina and Australia showing peaks between September and October. In Canada and Japan (the northernmost countries included in our study), the peak was observed in late spring (April–May), while in lower latitude Northern Hemisphere countries (e.g., Mexico and the United Arab Emirates), the peak typically occurred somewhat earlier (late February–March). Within the intertropical belt (intertropical belt), the typical timing of the primary epidemic peak was more variable across countries, as illustrated in the plots (Figures 1 and 2). As presented in Table 2, several countries (particularly in the ITB) experienced notable changes in the typical timing of the primary peak when comparing the pre‐COVID period (2016–2019) with the post‐COVID period (2021 onwards). The most significant shifts occurred in Argentina (September to June), Paraguay (August–September to June) and Brazil (March to October) (Table 2).

**TABLE 1:** Month, amplitude and duration of the primary seasonal hMPV peak by country, with global values and period‐specific ranges (2016–2024).

Country	Latitude	2016–2024			
		N. season with ≥ 50 reported hMPV cases	Duration in weeks (75% AAP), range (median)	Month primary peak	Amplitude primary peak (%)
Chile	−37.7	9 (2016–2024)	14–32 (16)	8.1 (Sep)	97.1%
Argentina	−35.4	8 (2016–2019, 2021–2024)	7–17 (15)	8.5 (Sep)	106.5%
Australia	−25.7	9 (2016–2024)	9–35 (18)	8.8 (Sep)	82.5%
Paraguay	−23.2	7 (2016–2019, 2022–2024)	8–29 (15)	5.7 (Jun)	98.5%
Brazil	−10.8	6 (2017–2019, 2022–2024)	14–22 (19.5)	3.0 (Mar‐Apr)	73.9%
Colombia	3.9	7 (2016–2019, 2022–2024)	31–41 (33)	5.5 (Jun)	54.3%
Panama	8.5	7 (2016–2019, 2022–2024)	15–27 (18)	8.5 (Sep)	88.0%
Thailand	15.1	3 (2022–2024)	22–42 (36)	1.4 (Feb)	83.9%
Guatemala	15.7	6 (2016–2017, 2019, 2022–2024)	15–31 (17.5)	7.7 (Aug)	91.9%
Hong Kong	22.4	6 (2018–2020, 2022–2024)	5–24 (21)	3.7 (Apr)	92.6%
Mexico	23.9	6 (2017–2018, 2021–2024)	13–23 (18.5)	1.8 (Feb)	102.4%
United Arab Emirates	23.9	4 (2021–2024)	25–29 (28)	2.6 (Mar)	93.3%
Qatar	25.3	8 (2016–2019, 2021–2024)	19–30 (23)	2.5 (Mar)	89.2%
Japan	37.6	7 (2016–2019, 2022–2024)	17–38 (26)	3.2 (Apr)	76.2%
Canada	61.4	8 (2016–2019, 2021–2024)	15–36 (20)	3.5 (Apr)	92.7%

**TABLE 2:** Comparison of hMPV epidemic timing, duration and amplitude before and after the COVID‐19 pandemic (selected countries, 2016–2019 vs. 2021–2024).

Country	Latitude	2016–2019			2021–2024		
		Duration in weeks (75% AAP), range	Month primary peak	Amplitude primary peak (%)	Duration in weeks (75% AAP), range	Month primary peak	Amplitude primary peak (%)
Chile	−37.7	14–17	8.2 (Sep)	95.9%	14–18	8.0 (Aug‐Sep)	100.2%
Argentina	−35.4	14–17	8.3 (Sep)	101.7%	7–16	5.5 (Jun)	103.7%
Australia	−25.7	17–25	8.9 (Sep)	89.4%	9–35	8.6 (Sep)	73.9%
Paraguay	−23.2	15–29	8.0 (Aug‐Sep)	96.7%	8–15	5.3 (Jun)	102.9%
Brazil	−10.8	18–22	2.9 (Mar)	81.1%	14–21	9.1 (Oct)	99.4%
Colombia	3.9	31–41	1.2 (Feb)	32.4%	32–36	5.5 (Jun)	64.9%
Panama	8.5	16–20	9.3 (Oct)	96.6%	15–27	7.4 (Aug)	68.4%
Guatemala	15.7	15–31	8.9 (Sep)	100.3%	17–22	7.3 (Aug)	91.5%
Qatar	25.3	19–30	2.5 (Mar)	92.3%	23–25	2.7 (Mar)	88.8%
Japan	37.6	22–38	3.1 (Apr)	79.5%	17–33	3.4 (Apr)	77.3%
Canada	61.4	19–20	3.2 (Apr)	93.4%	15–26	3.6 (Apr)	93.6%

**FIGURE 3:**
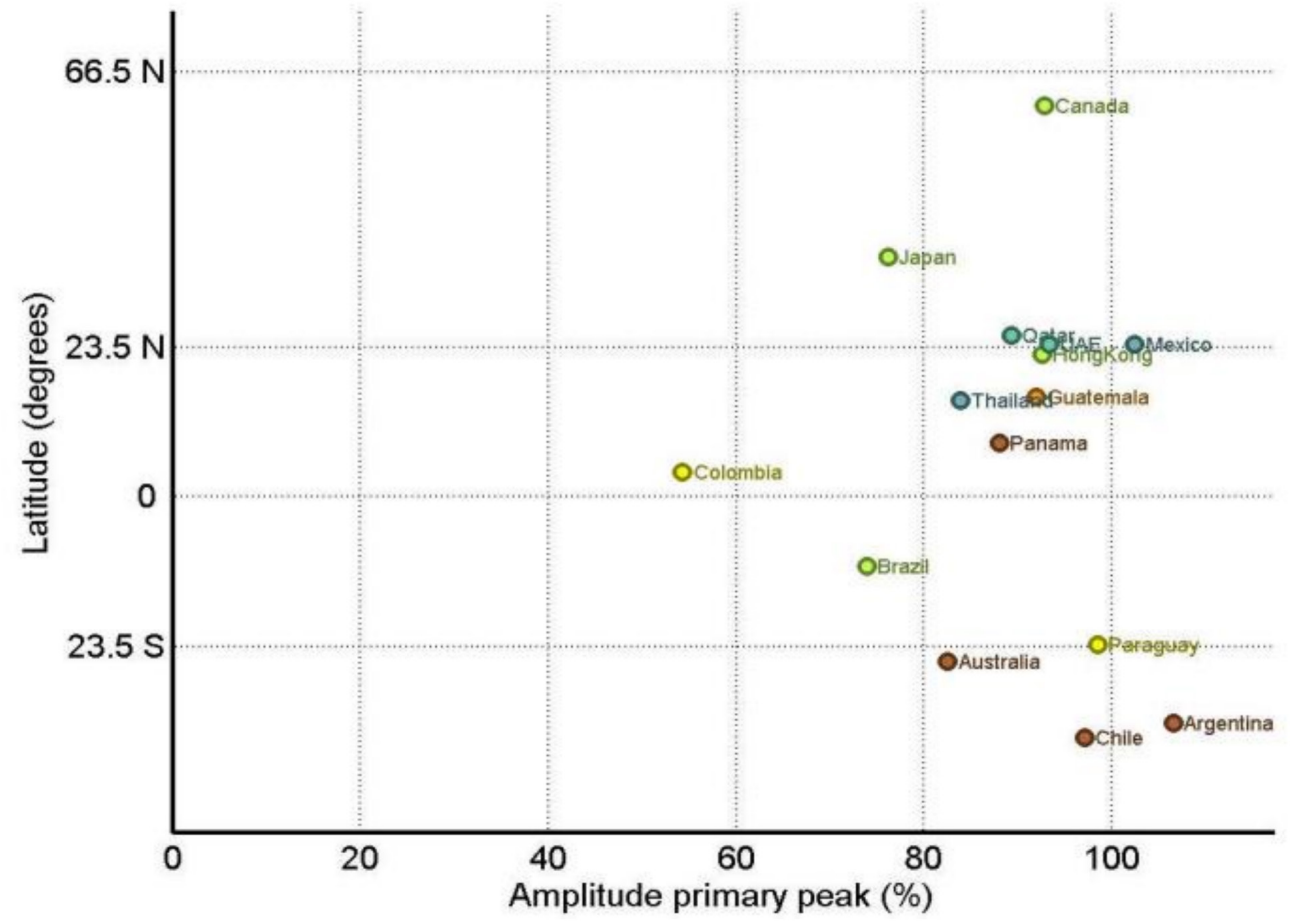
Amplitude of the primary peak of hMPV epidemics by country latitude. WHO FluNet, 2016–2025.

The amplitude of the primary seasonal peak also differed considerably between countries, highlighting the varying levels of hMPV activity throughout the year (Table 1 and Figures 1 and 3). Low‐amplitude seasonal patterns (less than 80% of the primary peak amplitude)—indicating a less distinct peak—were noted in Brazil, Colombia and Japan. In contrast, the most high‐amplitude patterns (> 95%) were evident in Argentina, Mexico, Paraguay and Chile. Among temperate countries in the Southern Hemisphere, Australia showed a moderate to high peak amplitude (82.5%). In countries with data for both periods (Table 2), the peak amplitude increased in some countries (e.g., Brazil and Colombia), decreased in others (e.g., Australia, Panama and Guatemala) and remained substantially stable in the remaining countries, with no apparent consistent trend across latitudes.

The median duration of hMPV epidemic was 19.5 weeks, with an overall range spanning from 5 to 42 weeks (Table 1). The duration varied widely across countries, with country‐specific median values ranging from 15 weeks in Argentina and Paraguay to as long as 36 weeks in Thailand (Table 1). At the extremes, Hong Kong showed the shortest minimum duration (5 weeks), while Thailand recorded the longest maximum duration (42 weeks). When analysed by latitude, epidemic durations had median values of 19.5 weeks in the intertropical belt (range 15–36), 23 weeks in the Northern Hemisphere (18.5–28) and 16 weeks in the Southern Hemisphere (15–18). After the COVID‐19 pandemic, the duration of hMPV epidemics showed remarkable differences compared with the pre‐pandemic period, although once again, this varied greatly by country. The duration of hMPV epidemics appears to have shortened most in Argentina (the range changed from 14–17 to 7–16 weeks) and in Japan (from 22–38 to 17–33 weeks). Conversely, other regions, such as Canada (from 19–20 to 15–26 weeks) and Panama (from 16–20 to 15–27 weeks), appear to experience extended epidemics in the postpandemic period. Of note, there was invariably some overlap in the distribution of epidemic durations in the pre‐pandemic and postpandemic periods, most likely due to the limited number of seasons included in the analysis. Finally, yet other countries like Chile and Colombia maintained a relatively stable duration, highlighting a complex and non‐uniform impact of the pandemic on hMPV seasonality.

## 4 Discussion

In our study, we aimed to investigate the timing of hMPV circulation in countries around the world, using data from the GISRS/WHO Flunet database. We showed how hMPV mostly follows the latitude‐aligned calendar typical of other respiratory viruses (autumn peaks in the Southern Hemisphere, late winter to early spring peaks in the Northern Hemisphere and more variable timing across the intertropical belt). hMPV is characterized by epidemics with a median duration of about 19.5 weeks, but with large diversity between countries, ranging between 5 and 42 weeks. Using a standardized approach applied to FluNet data from Week 1/2016 to Week 26/2025, and with due precautions in interpreting data based on a still limited number of seasons (especially in the postpandemic period), we suggested that the COVID‐19 pandemic period may have caused timing dislocations and hemisphere‐specific changes in amplitude, features that may bear direct implications for surveillance cadence and future prevention strategies. By extending observation through mid‐2025, this study provides one of the first comprehensive descriptions of hMPV seasonality in the post‐COVID era, an important step for understanding how global patterns are stabilizing after pandemic disruptions and for informing preparedness and prevention strategies.

Our results detail a neat latitude gradient in peak timing, with concrete country‐level expressions. In Southern Hemisphere temperate settings, peaks clustered in autumn (Chile and Argentina) or late winter–spring (Australia). In higher latitude Northern settings, spring peaks predominated (Canada and Japan in April), while lower latitude Northern countries peaked earlier (Mexico in February; Qatar and the United Arab Emirates in March–April). Within the intertropical belt, timing was more variable, with peaks distributed from February to September or hardly defined at all (e.g., in Colombia). These internal patterns mirror a previous large global synthesis of hMPV epidemics timing [16], and they align with national analyses in the United States, where hMPV typically peaks in late March with spring offsets [17], and with Australian series describing spring peaks [18, 19]. This heterogeneity, even among countries at comparable latitudes, likely reflects a complex interplay between ecological and surveillance‐related factors; differences in testing intensity, health‐system resilience and viral interference with RSV and influenza circulation may contribute to the observed variability beyond geographic latitude alone. Concerning duration, epidemics were shortest in the Southern Hemisphere (~16 weeks), intermediate in the intertropical belt (~19.5 weeks) and longest in the Northern Hemisphere (~23 weeks). These long, geographically structured windows imply sustained pressure that extends beyond a brief winter surge. The duration profile matches the ≈4.8‐month average reported across temperate and tropical sites in Li et al.'s work [16] and sits alongside US medians near 21 weeks in pre‐pandemic seasons with a well‐documented long season in 2021–2022 followed by a return towards baseline length in 2022–2024 [17]. Several biological and environmental factors may help explain the prolonged activity of hMPV (compared with other respiratory viruses, e.g., influenza). The various viral genotypes (A and B) and sublineages (A1, A2, B1, B2 and A2b) often cocirculate and can undergo dynamic replacement [20], which favours reinfections across antigenic groups. Environmental factors also impact hMPV activity, for example, epidemics may overlap monsoon seasons in the tropics, driven by rainfall, temperature and humidity [16]. In this context, ongoing climate change could also influence hMPV seasonality by altering local temperature and humidity patterns over time, a hypothesis that would require long‐term integration of meteorological and virological datasets to be formally tested.

Our results showed the COVID‐19 pandemic produced some instability in both peak timing and amplitude of hMPV epidemics, but this was not uniform across geographies. Timing shifts between the pre‐pandemic (2016–2019) and postpandemic (2021–2024) periods were recurrent and could be very large (2–3 months or more) but did not affect all countries and need confirmation in future investigations as they are still based on a limited number of seasons. These signals also accord with postpandemic appraisals describing sustained irregularities in hMPV seasonality and later hMPV than RSV peaks in temperate zones, especially in 2022–2023 and 2023–2024 [21], and they complement the US profile of re‐emergent spring peaks after 2021–2022 [17]. Our design was not intended to assign determinants causing changes in seasonality; observed shifts plausibly reflect a combination of public health measures and their relaxation, altered contact patterns and other persistent social changes, cocirculation and interference among respiratory viruses and changes in testing intensity. However, disentangling these factors requires data beyond FluNet's current fields: We therefore treat dislocations in seasonality as real constraints that planners must accommodate while surveillance stabilizes.

This study has some strengths. In particular, three design choices improved comparability between countries and therefore reduced bias: (1) the use of a uniform analytical framework (Fourier decomposition‐based method for peak timing, 75% AAP for duration), (2) applied conservative thresholds to calculate robust estimates and (3) excluded 2020 to avoid anchoring estimates to pandemic anomalies. In countries reporting more than one surveillance data type, we selected the one with the most detections, consistent with previously validated practices [13]. Another key strength of this study lies in the timeliness of the data, which extends up to July 2025 and therefore provides one of the most up‐to‐date assessments currently available. A number of limitations must be acknowledged as well. FluNet does not report denominators for hMPV testing: Positivity rates and burden cannot therefore be derived, and peak amplitude should not be read as intensity; moreover, low detections may stem from reduced testing intensity rather than reduced circulation. Surveillance sources are heterogeneous and case mix may therefore shift over time. Several regions remain underrepresented, notably the WHO European Region, as direct reporting to FluNet is not available and only aggregated data via ECDC are forwarded, limiting generalizability [13]. Residual COVID‐19 measures in 2021–2022 may have biased timing estimates, so early postpandemic results should be interpreted with caution. Moreover, although we extended observation to the epidemic Week 26/2025, the footprint of pandemic‐era dislocation likely varies by country and will certainly take additional seasons to stabilize into consistent seasonal patterns. Finally, the postpandemic period currently spans only three to four complete epidemic cycles, and interannual variability may still reflect transitional dynamics rather than stable trends; also, differential diagnostic priorities across years, such as temporary emphasis on SARS‐CoV‐2 or RSV testing, may have indirectly influenced the number of hMPV detections reported to FluNet. Notwithstanding these constraints, the alignment with global syntheses [16], national benchmarks [17] and regional series from Australia [18, 19] and the tropics [22, 23] supports the validity of our timing and duration estimates.

The implications of our findings are immediate and of great public health importance, considering that hMPV accounts for 5%–7% of paediatric hospitalizations for acute respiratory infections and contributes to morbidity among older adults and immunocompromised patients [24, 25]. To map the seasonality pattern of hMPV, more robustly surveillance programmes need to calibrate sampling, assay procurement and reporting cadence to latitude‐specific peak months and sustain coverage across at least 5 months rather than brief winter intervals. Also, current surveillance case definitions such as acute respiratory infection and in particular influenza like‐illness may not be adequate in capturing hMPV infections, because they often present with milder or atypical symptoms; slightly widening these definitions or integrating complementary clinical indicators could improve hMPV detection and strengthen surveillance representativeness. Forecasting models can use peak timing and duration estimates as priors, stratified by latitude and refreshed annually. For vaccine developers and policy makers—once hMPV or combination products are available—trial design, immunogenicity sampling and deployment strategies should account for later peaks than RSV in temperate regions and more heterogeneous calendars in the tropics and must necessarily include constant monitoring of trends in the postpandemic period to adapt immunization schedules to any changes in the epidemiological situation. Critically, reliance on surge capacity limited to a short winter peak is likely to miss a large share of infections; this underscores the need for surveillance systems that maintain testing capacity throughout extended periods of viral activity, which can vary substantially across countries and seasons. Our findings also underline the existing challenges in estimating the burden of disease of hMPV, overall and in different age groups, which were already highlighted by other authors in recent years, for instance, by Wang et al. for children under 5 years of age [26] and by Kulkarni et al. for older adults (≥ 65 years) [27]. Two key improvements would enhance the interpretability of global surveillance: the inclusion of denominators to enable positivity‐based intensity measures and harmonized classification of sentinel versus nonsentinel data to track changes in case mix. Moreover, routine inclusion of hMPV testing in diagnostics would be warranted also because hMPV symptoms are clinically indistinguishable from those caused by influenza, rhinovirus or RSV. These upgrades would allow future analyses to pair stable timing estimates with credible measures of epidemic magnitude and reduce uncertainty about the trajectory of renormalization.

In conclusion, the salient aspects of global seasonality of hMPV epidemics that we highlighted here are of great public health relevance to investigate trends in hMPV seasonality globally, but at the same time, they also need to be re‐evaluated carefully and regularly in the coming years to verify any persistent trend or unexpected fluctuations. Making careful use of updated, global epidemiological data are critical in the attempt to maximize the effectiveness of any future preventive strategies (including vaccines currently under development [28, 29, 30] as well as for preparedness purposes).

## Author Contributions

**Enrica Castellana:** conceptualization, methodology, writing – original draft, writing – review and editing. **Alessandra Picelli:** conceptualization, methodology, writing – original draft, writing – review and editing. **Emma Papini:** validation, writing – review and editing. **Guglielmo Bonaccorsi:** validation, writing – review and editing. **Jojanneke van Summeren:** validation, writing – review and editing. **Marco Del Riccio:** conceptualization, methodology, supervision, project administration, writing – original draft, writing – review and editing. **Saverio Caini:** conceptualization, methodology, supervision, project administration, writing – original draft, writing – review and editing.

## Funding

The authors have nothing to report.

## Ethics Statement

The authors have nothing to report.

## Conflicts of Interest

J.S. declare that Nivel has received collaborative grants from Sanofi, AstraZeneca and GSK outside the submitted work and that Nivel has received a grant from the Preparing for RSV Immunisation and Surveillance in Europe (PROMISE) project of the ‘Innovative Medicines Initiative 2 Joint Undertaking’ Grant Agreement No. 101034339. This Joint Undertaking gets support from the ‘European Union's Horizon 2020 research and innovation programme’ and the ‘European Federation of Pharmaceutical Industries and Associations’. M.D.R. reports having collaborated on research projects related to the epidemiology of influenza, RSV and SARS‐CoV‐2, funded by, or having provided consultancy services to, or received research or travel grants from AstraZeneca, GSK, CSL Seqirus, Moderna, MSD, Pfizer and Sanofi. The other authors have no competing interests to report.

## Supporting information


**Figure S1:** Time series of human metapneumovirus circulation in Chile. WHO FluNet, 2016–2025.
**Figure S2:** Time series of human metapneumovirus circulation in Argentina. WHO FluNet, 2016–2025.
**Figure S3:** Time series of human metapneumovirus circulation in Australia. WHO FluNet, 2016–2025.
**Figure S4:** Time series of human metapneumovirus circulation in Paraguay. WHO FluNet, 2016–2025.
**Figure S5:** Time series of human metapneumovirus circulation in Brazil. WHO FluNet, 2016–2025.
**Figure S6:** Time series of human metapneumovirus circulation in Colombia. WHO FluNet, 2016–2025.
**Figure S7:** Time series of human metapneumovirus circulation in Panama. WHO FluNet, 2016–2025.
**Figure S8:** Time series of human metapneumovirus circulation in Thailand. WHO FluNet, 2016–2025.
**Figure S9:** Time series of human metapneumovirus circulation in Guatemala. WHO FluNet, 2016–2025.
**Figure S10:** Time series of human metapneumovirus circulation in Hong Kong. WHO FluNet, 2016–2025.
**Figure S11:** Time series of human metapneumovirus circulation in Mexico. WHO FluNet, 2016–2025.
**Figure S12:** Time series of human metapneumovirus circulation in United Arab Emirates. WHO FluNet, 2016–2025.
**Figure S13:** Time series of human metapneumovirus circulation in Qatar. WHO FluNet, 2016–2025.
**Figure S14:** Time series of human metapneumovirus circulation in Japan. WHO FluNet, 2016–2025.
**Figure S15:** Time series of human metapneumovirus circulation in Canada. WHO FluNet, 2016–2025.
**Table S1:** Global circulation of human metapneumovirus in countries lying in the Northern hemisphere, intertropical belt or Southern hemisphere. WHO FluNet, 2016–2025.
**Table S2:** Global circulation of human metapneumovirus in countries belonging to the different WHO regions. WHO FluNet, 2016–2025.
**Table S3:** Global circulation of human metapneumovirus by season. WHO FluNet, 2016–2025.

## Data Availability

The data that support the findings of this study are available in WHO FluNet at https://www.who.int/tools/flunet. These data were derived from the following resources available in the public domain: WHO FluNet, https://www.who.int/tools/flunet.
