# Supplementary material for: Human Metapneumovirus Circulation and Seasonality on a Global Scale, 2016–2025: Changes in Patterns and Epidemic Timing in the Pre‐ Versus Post‐COVID‐19 Era
Source: Influenza Other Respir Viruses. 2025 Dec 17;19(12):e70200. doi: 10.1111/irv.70200 (PMC12981517; doi:10.1111/irv.70200)
Supplement: Supplementary file 1 — Figure S1: Time series of human metapneumovirus circulation in Chile. WHO FluNet, 2016–2025. Figure S2: Time series of human metapneumovirus circulation in Argentina. WHO FluNet, 2016–2025. Figure S3: Time series of human metapneumovirus circulation in Australia. WHO FluNet, 2016–2025. Figure S4: Time series of human metapneumovirus circulation in Paraguay. WHO FluNet, 2016–2025. Figure S5: Time series of human metapneumovirus circulation in Brazil. WHO FluNet, 2016–2025. Figure S6: Time series of human metapneumovirus circulation in Colombia. WHO FluNet, 2016–2025. Figure S7: Time series of human metapneumovirus circulation in Panama. WHO FluNet, 2016–2025. Figure S8: Time series of human metapneumovirus circulation in Thailand. WHO FluNet, 2016–2025. Figure S9: Time series of human metapneumovirus circulation in Guatemala. WHO FluNet, 2016–2025. Figure S10: Time series of human metapneumovirus circulation in Hong Kong. WHO FluNet, 2016–2025. Figure S11: Time series of human metapneumovirus circulation in Mexico. WHO FluNet, 2016–2025. Figure S12: Time series of human metapneumovirus circulation in United Arab Emirates. WHO FluNet, 2016–2025. Figure S13: Time series of human metapneumovirus circulation in Qatar. WHO FluNet, 2016–2025. Figure S14: Time series of human metapneumovirus circulation in Japan. WHO FluNet, 2016–2025. Figure S15: Time series of human metapneumovirus circulation in Canada. WHO FluNet, 2016–2025. Table S1: Global circulation of human metapneumovirus in countries lying in the Northern hemisphere, intertropical belt or Southern hemisphere. WHO FluNet, 2016–2025. Table S2: Global circulation of human metapneumovirus in countries belonging to the different WHO regions. WHO FluNet, 2016–2025. Table S3: Global circulation of human metapneumovirus by season. WHO FluNet, 2016–2025. [file IRV-19-e70200-s001.docx]

**Supplementary Figure S1**: Time-series of human metapneumovirus circulation in Chile. WHO FluNet, 2016-2025.

**Supplementary Figure S2**: Time-series of human metapneumovirus circulation in Argentina. WHO FluNet, 2016-2025.

**Supplementary Figure S3**: Time-series of human metapneumovirus circulation in Australia. WHO FluNet, 2016-2025.

**Supplementary Figure S4**: Time-series of human metapneumovirus circulation in Paraguay. WHO FluNet, 2016-2025.

**Supplementary Figure S5**: Time-series of human metapneumovirus circulation in Brazil. WHO FluNet, 2016-2025.

**Supplementary Figure S6**: Time-series of human metapneumovirus circulation in Colombia. WHO FluNet, 2016-2025.

**Supplementary Figure S7**: Time-series of human metapneumovirus circulation in Panama. WHO FluNet, 2016-2025.

**Supplementary Figure S8**: Time-series of human metapneumovirus circulation in Thailand. WHO FluNet, 2016-2025.

**Supplementary Figure S9**: Time-series of human metapneumovirus circulation in Guatemala. WHO FluNet, 2016-2025.

**Supplementary Figure S10**: Time-series of human metapneumovirus circulation in Hong Kong. WHO FluNet, 2016-2025.

**Supplementary Figure S11**: Time-series of human metapneumovirus circulation in Mexico. WHO FluNet, 2016-2025.

**Supplementary Figure S12**: Time-series of human metapneumovirus circulation in United Arab Emirates. WHO FluNet, 2016-2025.

**Supplementary Figure S13**: Time-series of human metapneumovirus circulation in Qatar. WHO FluNet, 2016-2025.

**Supplementary Figure S14**: Time-series of human metapneumovirus circulation in Japan. WHO FluNet, 2016-2025.

**Supplementary Figure S15**: Time-series of human metapneumovirus circulation in Canada. WHO FluNet, 2016-2025.

**Supplementary Table S1**: Global circulation of human metapneumovirus in countries lying in the Northern hemisphere, inter-tropical belt, or Southern hemisphere. WHO FluNet, 2016-2025.

| **Geographical area** | **N. of country-seasons** | **n. of hMPV detections reported to Flunet** | **Median detections per season** | **N (%) country-seasons with 1-24 reported cases** | **N (%) country-seasons with 25-49 reported cases** | **N (%) country-seasons with ≥ 50 reported cases** |
| --- | --- | --- | --- | --- | --- | --- |
| Northern hemisphere | 61 | 60,609 | 137 | 18 (29.5%) | 4 (6.6%) | 39 (63.9%) |
| Inter‑tropical belt | 106 | 34,717 | 24 | 83 (50.3%) | 19 (11.5%) | 63 (38.2%) |
| Southern hemisphere | 38 | 49,831 | 801 | 7 (18.4%) | 1 (2.6%) | 30 (79.0%) |
| **Total** | **205** | **145,157** | **49** | **108 (40.9%)** | **24 (9.1%)** | **132 (50.0%)** |

**Supplementary Table S2**: Global circulation of human metapneumovirus in countries belonging to the different WHO regions. WHO FluNet, 2016-2025.

| **WHO region** | **N. of hMPV detections reported to Flunet** | **Median detections per season** | **N (%) seasons with 1-24 reported cases** | **N (%) seasons with 25-49 reported cases** | **N (%) seasons with ≥ 50 reported cases** |
| --- | --- | --- | --- | --- | --- |
| African Region (AFR) | 320 | 10 | 9 (69.2%) | 1 (7.7%) | 3 (23.1%) |
| Region of the Americas (AMR) | 100,643 | 38 | 65 (42.5%) | 15 (9.8%) | 73 (47.7%) |
| Eastern Mediterranean (EMR) | 8,265 | 94 | 8 (33.3%) | 0 (0.0%) | 16 (66.7%) |
| European Region (EUR) | 0 | - | - | - | - |
| South‑East Asia (SEAR) | 1,781 | 19 | 10 (52.6%) | 2 (10.5%) | 7 (36.9%) |
| Western Pacific (WPR) | 34,148 | 89 | 16 (29.1%) | 6 (10.9%) | 33 (60.0%) |
| **Total** | **145,157** | **49** | **108 (40.9%)** | **24 (9.1%)** | **132 (50.0%)** |

**Supplementary Table S3**: Global circulation of human metapneumovirus by season. WHO FluNet, 2016-2025.

| **Season** | **N. of hMPV detections reported to Flunet** | **Median detections per season** | **N (%) seasons with 1-24 reported cases** | **N (%) seasons with 25-49 reported cases** | **N (%) seasons with ≥ 50 reported cases** |
| --- | --- | --- | --- | --- | --- |
| 2016 | 9,292 | 35 | 9 (34.6%) | 5 (19.2%) | 12 (46.2%) |
| 2017 | 12,953 | 55 | 13 (40.6%) | 3 (9.4%) | 16 (50.0%) |
| 2018 | 15,445 | 28 | 17 (50.0%) | 3 (8.8%) | 14 (41.2%) |
| 2019 | 12,351 | 59 | 10 (38.5%) | 2 (7.7%) | 14 (53.8%) |
| 2020 | 919 | 9 | 16 (66.6%) | 4 (16.7% | 4 (16.7% |
| 2021 | 5,006 | 21 | 11 (55.0%) | 2 (10.0%) | 7 (35.0%) |
| 2022 | 26,353 | 151 | 6 (20.0%) | 2 (6.7%) | 22 (73.3%) |
| 2023 | 29,024 | 168 | 12 (34.3%) | 1 (2.8%) | 22 (62.9) |
| 2024 | 33,814 | 90 | 14 (37.8%) | 2 (5.4%) | 21 (56.8%) |
| **Total** | **145,157** | **49** | **108 (40.9%)** | **24 (9.1%)** | **132 (50.0%)** |
